# An anterolateral dislocated monteggia lesion with plastic lateral bowing of the ulna associated with ipsilateral epiphyseal fracture of the distal radius: a case report with 4 years of follow-up

**DOI:** 10.1080/23320885.2021.2024762

**Published:** 2022-01-27

**Authors:** Mitsuhiko Takahashi, Ryo Miyagi, Hirofumi Kosaka, Hiroshi Egawa

**Affiliations:** Department of Orthopaedic Surgery, Tokushima Prefectural Central Hospital, Tokushima, Japan

**Keywords:** Monteggia lesion, plastic deformity, epiphyseal fracture

## Abstract

We encountered a rare presentation of anterolateral dislocation of the radial head with plastic lateral bowing of the ulna associated with ipsilateral epiphyseal fracture of the distal radius in a child. The patient was treated surgically and reached skeletal maturity 4 years later with no functional or growth deficiency.

## 1. Introduction

A Monteggia lesion is a dislocation of the radial head associated with a fracture of the ulnar shaft. Bado classified Monteggia lesions into four types based mainly on the direction of dislocation of the radial head [1]. He also described Monteggia equivalents, which are mostly the same as type I Monteggia lesions with anterior dislocation of the radial head. Bado also described a type II equivalent that is only seen with epiphyseal fracture of the dislocated radial head and stated that type III and type IV had no equivalents. Confusion persists regarding the definition of Monteggia equivalents. A dislocated radial head associated with plastic deformity of the ulna is common in the skeletally immature population and could be classified as a Monteggia lesion. Here, we describe a previously unreported Monteggia variant that presented as anterolateral dislocation of the radial head and plastic lateral bowing of the ulna associated with ipsilateral epiphyseal fracture of the distal radius. In addition, we discuss Monteggia lesions and its equivalents, and review the literature.

## 2. Case report

**Figure 1.**
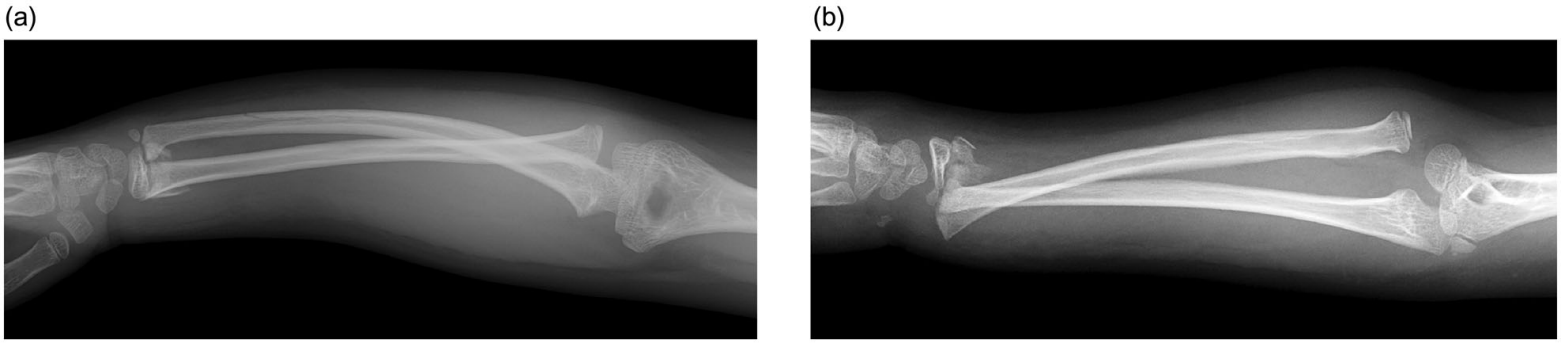
(a, b) Radiographs of the right forearm at the first presentation. Note that true lateral view was not obtained at this point.

**Figure 2.**
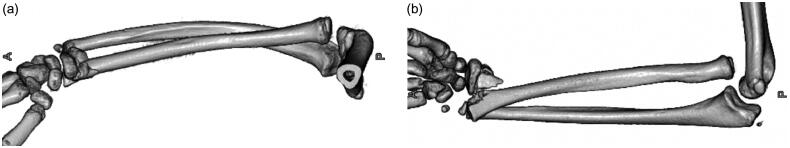
Anteroposterior (a) and lateral (b) views of reconstructed three-dimensional computed tomography (CT) of the right forearm. Anteroposterior and lateral directions of the forearm were adjusted according to the elbow joint orientation in this CT and later radiographs.

An 8-year girl, who was otherwise healthy, presented to our emergency room with pain and functional deficit in the right forearm. She was staying at a lodging facility without her parents while attending a summer cram school. She explained that right forearm hurt when she woke up in the morning. A staff member accompanying her reported that she had not fallen out of bed or fallen down in the lodging facility. On examination, there was swelling of the forearm and lateral aspect of the elbow, as well as marked restriction of flexion/extension at the elbow and supination/pronation of the forearm. There were no signs of a neurovascular deficit in the right arm, and moderate finger movements were observed. Radiographs showed plastic deformity of the right ulnar diaphysis and anterolateral dislocation of the radial head (Figure 1). There was also a Salter-Harris type II epiphyseal injury of the ipsilateral distal radius with a relatively large metaphyseal fragment. Reconstructed three-dimensional computed tomography (CT) adjusted according to the elbow joint orientation revealed plastic deformity of the ulna with lateral bowing (Figure 2).

**Figure 3.**
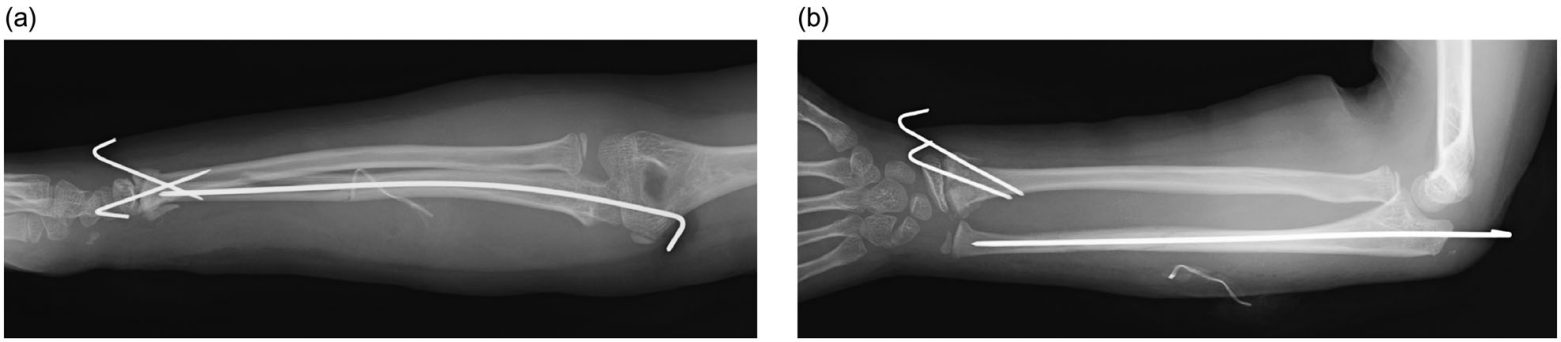
Anteroposterior (a) and lateral (b) views of radiographs at immediately after surgery.

**Figure 4.**
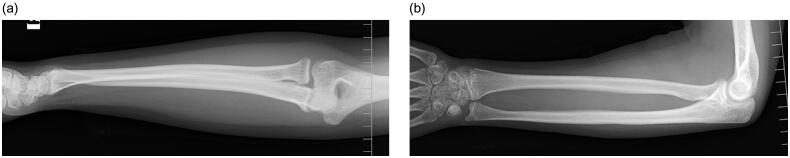
Anteroposterior (a) and lateral (b) views of radiographs 4 years after surgery.

Surgery was performed under general anesthesia on the same day. We first attempted closed reduction of the bowed ulna by pushing the midshaft of the ulna in the medial direction but were unsuccessful. Reduction was then achieved by directly dragging the bent midshaft of the ulna using a blunt single-prong bone hook *via* a skin incision over the ulnar shaft. Next, an intramedullary wire of diameter 2.0 mm was inserted *via* the olecranon, while physiologic lateral bowing of the proximal ulna was maintained (Figure 3). The dislocated radial head was spontaneously repositioned by reduction of the ulna. Fluoroscopic imaging confirmed resolution of the eccentric movement of the radial head during pronation/supination of the forearm. The relatively large diaphyseal fragment of the distal radius was reduced through a volar skin incision followed by percutaneous cross-pinning through the metaphysis. The anterior forearm fascia was released as much as possible through the skin incisions to minimize the risk of compartment syndrome. The arm was immobilized with the forearm in neutral rotation and the elbow joint at 90 degrees for 4 weeks. The wires were removed after confirmation of callus formation at both the ulna and radius. Four years later, the patient is 12 years of age with closure of all the physes around the elbow but not those at the distal radius or ulna. There are no signs of impaired growth of the physes, and joint motion at the elbow and wrist and forearm rotation were not restricted (Figure 4).

## 3. Discussion

The first description of Monteggia lesions was published by Monteggia in 1814 and concerned two adult cases of fracture of the proximal third of the ulna with conjoined anterior dislocation of the proximal radius [2]. In 1967, Bado devised a classification system for Monteggia lesions based on the direction of dislocation of the radial head [1]. Using this system, the most frequent presentation (in approximately 60% of cases) is type I, namely, an anteriorly dislocated radial head and ulnar fracture with anterior bowing. The likely mechanism of this injury is a fall on an outstretched arm with forced pronation caused by external rotation of the proximal upper arm and trunk [3,4]. This forced pronation leads to anterior dislocation of the proximal radius with the forearm fixed in pronation clinically and radiographically. Subsequently, Bado described type I Monteggia equivalents, which are similar lesions that occur *via* the same mechanism, and other studies have supported this idea [1,5]. Debate persists regarding whether Monteggia dislocation-fracture caused by the forced pronation mechanism can be all classified as Bado type I. Seven years before Bado’s paper, Fahey described a Monteggia equivalent that presented as a dislocated radial head without fracture of the ulna but with bending [6], which denoted plastic deformity of the ulna. Letts et al. later proposed a new classification for Monteggia lesions in skeletally immature children that included plastic deformity and greenstick fracture of the ulna [7]. Although the Letts system is generally appropriate for classification of Monteggia lesions in children, plastic deformity and greenstick fracture of the ulna were described only for anterior bends, just like Bado type I.

In our case, the ulna was bent laterally based on the oriented 3D-CT, which was, strictly speaking, unclassifiable in the Letts system. Direction of the dislocated radial head generally corresponds to that of the ulnar deformity. However, Cepelik et al. recently reported that dislocation of the radial head and plastic deformity of the ulna can occur in any direction with Monteggia lesions in children, and proposed that dislocation of the radial head associated with plastic deformity of the ulna was a ‘true’ Monteggia lesion and not a Monteggia equivalent [8].

Irrespective of the direction of dislocation and deformity, prompt diagnosis and early treatment with correction of the ulna fracture or deformity is essential. Manual reduction is beneficial for treatment of Monteggia lesions if they are treated early [9]. As a means of early diagnosis, the maximum ulnar bow is a valuable sign for detection of ulnar deformity [10] and is measured on a lateral plain radiograph. There have been reports of delayed or missed diagnoses, especially in patients with a plastic deformity of the ulna, that could have been avoided by careful radiographic interpretation [5,11–13]. A delayed or missed diagnosis would lead to sequelae in terms of the function of the forearm and/or need for a more invasive surgical procedure later on. However, it can be difficult to obtain precise anteroposterior and lateral plain radiographs for a child experiencing acute pain in the emergency department setting. These radiographs are not only critical for accurate diagnosis but also important for determining the direction of the force needed for correct repositioning of the ulna and radial head. In our patient, a precise lateral view of the affected forearm was not obtained (Figure 1). However, reconstructed three-dimensional CT adjusted to anteroposterior and lateral directions clearly revealed lateral bowing of the ulna and anterolateral dislocation of the radial head. Precisely oriented three-dimensional CT may be necessary to avoid a missed diagnosis and to decide on a treatment plan in many cases of Monteggia lesions and their equivalents.

A literature search identified five case reports describing combinations of various types of Monteggia lesions and ipsilateral epiphyseal fracture of the distal radius [14–18]. In all cases, the injury was associated with a fall from height and included an ulna fracture and an ipsilateral Salter-Harris type II lesion of the distal radius. The direction of dislocation of the radial head was anterior (Bado type I) in two cases, posterior (type II) in 1 case, and lateral (type III) in two cases. In all cases, open reduction was needed after failure of closed reduction. To our knowledge, this is the first report of a Monteggia lesion with plastic deformity of the ulna associated with ipsilateral epiphyseal fracture of the distal radius. The mechanism of this type of injury was not clear. Both Evans and Bado mentioned that type I and II lesions are often accompanied by lesions of the distal radius but neither provided any supporting data [1,3]. The likely cause of the lesion at the distal radius is a fall on an outstretched arm and the forced pronation mechanism may explain a Monteggia lesion or equivalent. Further research is needed to understand the mechanism of this type of injury.

In conclusion, an 8-year girl presented anterolateral dislocation of the radial head with plastic lateral bowing of the ulna associated with ipsilateral epiphyseal fracture of the distal radius. She was treated successfully by open reduction of the ulnar shaft and distal radius. She reached skeletal maturity 4 years later with clinically and radiologically satisfactory results. Diagnosis of a Monteggia lesion with plastic deformity may be delayed or missed. Careful examination using precisely oriented CT would be useful when deciding on a treatment strategy.

## Informed consent

Informed consent has been obtained from the patient and her family.
